# Dementia and migration: culturally sensitive healthcare services and projects in Germany: A scoping review

**DOI:** 10.1007/s00391-022-02022-w

**Published:** 2022-02-04

**Authors:** Jessica Monsees, Sümeyra Öztürk, Jochen René Thyrian

**Affiliations:** 1 Deutsches Zentrum für Neurodegenerative Erkrankungen e. V. Rostock/Greifswald, Ellernholzstr. 1–2, 17489 Greifswald, Germany; 2 Demenz Support Stuttgart, Stuttgart, Germany; 3 Institute for Community Medicine, University Medicine Greifswald, Greifswald, Germany

**Keywords:** Healthcare, Vulnerable Populations, Support, Federal States, Access, Gesundheitsversorgung, Vulnerable Bevölkerungsgruppen, Unterstützung, Bundesländer, Zugang

## Abstract

**Background:**

There are approximately 96,500 people with a migration background (PwM) with dementia in Germany. They and their families face not only dementia-related challenges but also the challenge of having little knowledge about the healthcare system and its services and thus more difficulty in accessing support. Germany’s national dementia strategy recognises these individuals as a risk group and thus aims to expand the provision of culturally sensitive information and healthcare services.

**Objective:**

To determine the amount of culturally sensitive information and healthcare services as well as projects on dementia and migration.

**Method:**

With a scoping review the PsycInfo, PsycArticles and Psychology & Behavioral Sciences Collection databases, Google Search, the network map (*Netzwerkkarte* on the website www.demenz-und-migration.de) and the websites of various research funding bodies were used to find culturally sensitive information and healthcare services as well as current projects on dementia and migration.

**Results:**

Listed are 45 care services as well as 3 additional projects that deal with dementia and migration at the local level. The geographical distribution of the offers shows that most of the services can be found in federal states where most PwM with dementia live.

**Discussion:**

It is necessary to provide information and healthcare services in all regions and to adapt them to PwM. Different aspects and culturally sensitive measures are important when informing PwM with dementia, as such information can enable these individuals to access the healthcare system and help to provide them with care. It is important to bring together relevant stakeholders to provide access and services that improve the situation of PwM with dementia and their families.

## Abstract (de)

**Hintergrund:**

In Deutschland leben etwa 96.500 Menschen mit Migrationshintergrund („people with a migration background“, PwM), die an Demenz erkrankt sind. Sie und ihre Familien stehen nicht nur vor demenzbedingten Herausforderungen, sondern auch vor dem Problem, dass sie wenig über das Gesundheitssystem und seine Angebote wissen und daher Schwierigkeiten haben, Unterstützung zu erhalten. Die nationale Demenzstrategie Deutschlands erkennt sie als Risikogruppe an und hat als Ziel, das Angebot an kultursensiblen Informationen und Versorgungsangeboten zu erweitern.

**Ziel:**

Ermittlung kultursensibler Informations- und Versorgungsangebote sowie von Projekten zum Thema Demenz und Migration.

**Methode:**

Im Rahmen eines Scoping-Reviews wurden die Datenbanken PsycInfo, PsycArticles und Psychology & Behavioral Sciences Collection, Google Search, die Netzwerkkarte auf der Website www.demenz-und-migration.de sowie die Websites verschiedener Forschungsförderer nach kultursensible Informations- und Versorgungsangeboten sowie aktuellen Projekten zu Demenz und Migration durchsucht.

**Ergebnisse:**

Aufgeführt sind 45 Versorgungsangebote sowie 3 weitere Projekte, die sich mit Demenz und Migration beschäftigen. Die geografische Verteilung der Ergebnisse zeigt, dass die Bundesländer, in denen die meisten PwM mit Demenz leben, auch die meisten Angebote bieten.

**Schlussfolgerung:**

Es ist nötig, in allen Regionen Informations- und Versorgungsangebote bereitzustellen und diese an PwM anzupassen. Bei der Aufklärung von PwM mit Demenz sind verschiedene kultursensible Maßnahmen und Aspekte wichtig, da solche Informationen PwM den Zugang zum Gesundheitssystem ermöglichen und bei ihrer Versorgung helfen können. Wichtig ist, relevante Interessenvertreter zu vernetzen, um den Zugang zu Angeboten zu arrangieren, die die Situation von PwM mit Demenz und ihren Familien verbessern.

In Germany, 1.99 million people with a migration background (PwM) are older than 64 years and approximately 96,500 are estimated to be living with dementia. The PwM with dementia and their families face not only dementia but also challenges related to accessing information and the healthcare system. For them to utilize services that provide information, care and support, these services have to be low-threshold, culturally sensitive and adapted to their needs. To help with that, projects on the topic are needed to gain knowledge about PwM with dementia and their needs in healthcare.

## Background

Over the course of its history, Germany has turned from an emigration country into an immigration country. The recruitment of guest workers from abroad in the mid-1900s, the collapse of the Soviet Union, the development of high levels of youth unemployment in southern Europe and the Syrian conflict have all contributed to a rise in Germany’s migrant population [10, 23]. According to census data, the number of PwM amounts to 18.6 million people, which accounted for 23% of the population in Germany in 2018 [27]. In Germany, as in many other countries of Europe, the number of PwM aged 65 years or older is increasing. A total of 1.99 million PwM are older than 64 years, which represents 11.52% of the German population who are older than 65 years. A growing probability of age-associated and chronic diseases such as dementia is a consequence of increasing age [17, 25]. An analysis from 2019 showed that there were approximately 96,500 PwM with dementia living in Germany at that time, with distinct regional differences regarding the number of PwM and their origin of migration. Most PwM with dementia reside in North-Rhine Westphalia (26,000), Baden-Wuerttemberg (18,100) and Bavaria (16,700), which is not surprising as they are the federal states where the greatest numbers of PwM can generally be found. Fewer PwM with dementia live in Mecklenburg-Western Pomerania (500), Thuringia (500), Saxony-Anhalt (650) and Brandenburg (650). The place of origin of most PwM with dementia (84,500) are other European countries, with most originating from Poland (14,000), Italy (8900), Turkey (8800), Romania (6400) and the Russian Federation (6300). Unpublished results from the abovementioned analysis reveal an uneven distribution across regions. Most people with dementia who originate from (a) Poland are found in North Rhine-Westphalia (6000) and Bavaria (1400), while (b) those from Italy reside in Baden-Wuerttemberg (2900) and North Rhine-Westphalia (2400), (c) those from Turkey live in North Rhine-Westphalia (3100) and Baden-Wuerttemberg (1300), (d) those from Russia live in North Rhine-Westphalia (1700) and Baden-Wuerttemberg (1000) and (e) those from Romania live in Bavaria (2500) and Baden-Wuerttemberg (2100) [19]. Despite the diversity of the group of PwM with dementia, there have been common challenges identified for this group and their families. It has been reported that there is a lack of culturally sensitive diagnostic and screening tools, which results in difficulties in diagnosing dementia [2, 22]. Additionally, culturally sensitive information and healthcare services are also missing for this population [4, 10, 16, 18]. The PwM show a lack of awareness and knowledge about the healthcare system, its services and support and how to utilize them [4, 16, 18]. Further complicating this situation are language problems that can arise in communication with healthcare professionals, service providers and others [4, 16] or false beliefs, such as thinking that dementia is a regular part of ageing [2, 24]. The PwM can be distrustful of the healthcare system and healthcare professionals or fear facing stigma or judgement from other people when utilizing formal help [10, 16, 18]. These factors are only just a few that play an important role in healthcare for PwM with dementia and their families. These circumstances are associated with worsened access to the healthcare system; thus, services have to be low-threshold, culturally sensitive and adapted to their needs [13, 29]. A more detailed overview of this topic is provided by Alzheimer Europe (2018) [1]. This identifies PwM with dementia as a group at special risk that needs appropriate care with culturally sensitive healthcare services and information that are adapted to their needs. However, the regional differences in areas where PwM with dementia live make it hard to implement a nationwide solution. Therefore, regional and local initiatives that are adapted to the situation on site also have to be established. Germany’s first national dementia strategy acknowledges PwM with dementia as a risk group and is committed to providing support and care for them. Therefore, the national dementia strategy aims to implement measures to support PwM with dementia. One measure is to expand the availability of culturally sensitive information and counselling services [14].

This scoping review provides an overview of existing national, regional and local healthcare services and information on where they are located and where action needs to be taken. In addition, this article depicts the current projects on dementia and migration present in Germany. This will help (a) affected people to see where they can find support and (b) service providers to initiate and offer new services and get in contact with each other.

## Method

For this analysis a scoping review was used. This type of review is designed to a) provide an overview of existing information, b) process information on a topic where not much scientific research is available and c) show gaps in that topic [28]. This approach is used to depict the spectrum of healthcare services and projects on dementia and migration in this analysis.

The websites of the Federal Ministry of Education and Research (BMBF), the Federal Ministry of Health (BMG), the Germany Research Foundation (DFG), and the German Aerospace Center (DLR) were consulted to see if they fund projects on dementia and migration. In addition, PsycInfo, PsycArticles, and Psychology and Behavioral Sciences Collection databases were screened via Ebscohost, which is an online reference tool offering the opportunity to search within a variety of databases. The databases used in this review were selected because they are able to deliver relevant literature on the thematic object of this article. These searches yielded no results and an internet search in Google was conducted on 12 and 13 January 2021. The search included the terms “*Projekt*” or “*Initiative*” in combination with “*Demenz*” or “*Alzheimer*”, “*Migration*” or “*Migrationshintergrund*” and “*Deutschland*” and was limited to websites from Germany in the period from 1 January 2016 to 31 December 2020. The results were in German and were included if a project was ongoing and specifically addressed dementia and migration. The results were excluded if a project had already ended and did not focus on dementia and migration. Projects on migration and health were excluded if they did not solely focus on dementia. Additionally, the German Alzheimer Society was consulted.

The DeMigranz project on dementia and migration connects stakeholders from the healthcare system, communities, migrant organizations and the government ministry to build networks. The aim is to establish culturally sensitive healthcare and information services in the federal states so that PwM with dementia and their families will be informed about dementia and included in the German healthcare system [6]. The “*Netzwerkkarte*” by DeMigranz lists available dementia-related services, organizations and projects that cater to PwM; it can be found at the following website: www.demenz-und-migration.de. The “*Netzwerkkarte*” was consulted on 15 October 2020 and 17 March 2021 for potential updates to determine how many services exist, their location and their type. Thus far, this is the only platform that offers an overview of dementia-related services and support for PwM; hence, it was chosen for this scoping review.

## Results

**Fig. 1:**
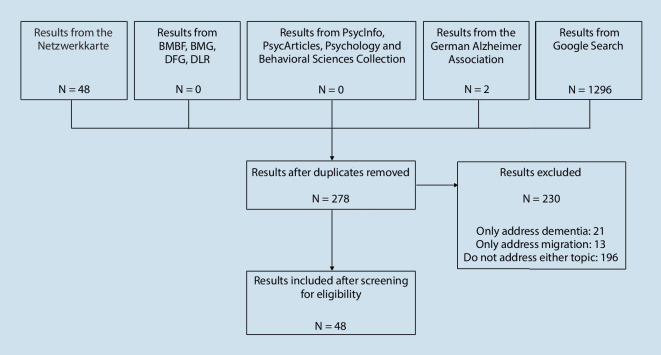
Flowchart showing the selection process for culturally sensitive healthcare and information services and projects. *BMBF* Federal Ministry of Education and Research, *BMG* Federal Ministry of Health, *DFG* Germany Research Foundation, *DLR* German Aerospace Center

The internet search yielded 1296 results, of which 1036 were duplicates. Of the remaining 260 results, 227 were excluded from the analysis because they did not address dementia and migration or only addressed one of these topics. Most of the suitable results (*n* = 30) from the remaining 33 are included in “*Netzwerkkarte*”. In the end, three additional projects on a federal state level emerged from the internet search for the analysis. The “*Netzwerkkarte*” lists 4 results on a national level and 44 on a federal state level. Of these 48 results, 45 are included in the analysis because they are projects, healthcare and information services aimed at PwM. The other three results represent partners of DeMigranz but do not offer services for PwM with dementia and were therefore excluded from the analysis [8]. The German Alzheimer Society reported two projects that operate nationwide and are included in the “*Netzwerkkarte*”. In the end, 48 culturally sensitive healthcare and information services and projects were included (see Fig. 1). Table 1 provides an overview of these services and projects.

**Table 1:** Overview on services, information and projects available for PwM with dementia in Germany

Location	Number	Type	Language(s)
Germany	4 (1 ended in 2019)	Counselling	Turkish, Russian, Italian, Greek, Serbo-Croat, Polish, Spanish, Arabic, Vietnamese
		Support services	
		Care services	
		Information	
		Networking	
North Rhine-Westphalia	12	Counselling	Turkish, Greek, Russian, multilingual
		Care services	
		Training	
Baden-Wurttemberg	7	Counselling	Turkish, Russian, Polish, Greek, Arabic, Croatian, Italian
		Information	
		Care services	
		Support services	
Bavaria	5	Counselling	Turkish, Russian, Arabic, Italian, Greek, Serbo-Croat, Polish, Spanish, Vietnamese
		Support services	
		Training	
Hesse	5	Counselling	Turkish, Russian
		Care services	
		Support services	
Berlin	4	Counselling	Turkish, Serbo-Croat, Spanish, Russian, Arabic, Vietnamese
		Support services	
Saxony	3	Counselling	Russian, Vietnamese
		Care services	
Hamburg	2	Care services	Turkish, multilingual
		Counselling	
Lower Saxony	2	Counselling	Turkish, Russian, Italian
Bremen	1	Counselling	Turkish
		Support service	
Saarland	1	Information	Turkish, Arabic, Russian, English, Italian
Schleswig-Holstein	1	Counselling	Turkish, Russian, Polish
Thuringia	1	Counselling	Russian, Arabic
Brandenburg, Mecklenburg-Western Pomerania, Rhineland-Palatinate, Saxony-Anhalt	/	/	/

### Germany

A total of four services were found that offer counselling in Turkish, Russian, Italian, Greek, Serbo-Croat, Polish, Spanish, Arabic, and Vietnamese, with one of them also offering support and care services in Russian.

One project ran until 2019 and aimed to inform PwM about dementia and possible support measures and motivated counsellors to provide more culturally sensitive services. One measure that was implemented was telephone counselling in Turkish, which is still offered. As a follow-up, a website (www.demenz-und-migration.de) was established in cooperation with DeMigranz to provide information about dementia in different languages (German, Turkish, Polish, Russian, English, Arabic), “*Netzwerkkarte*” and information to facilitate contact with PwM [7, 9]. Another project is DeMigranz, which is described in the “Method” section.

### North Rhine-Westphalia

A total of 12 offers could be identified, 7 provide counselling in Turkish, Greek and Russian and/or care services in Turkish, 2 projects train people who speak different languages to give information about dementia and related services, and 1 is a Russian apartment-sharing community [8]. Another project trains persons with different linguistic backgrounds to act as contact persons for PwM with dementia and their families, inform them about dementia and help them get in contact with healthcare services [11]. Another project qualifies people from different linguistic backgrounds to offer support services and information to PwM with dementia and their families [15].

### Baden-Wuerttemberg

A total of six services were detected that provide information, counselling, care services and care facilities in Turkish, Russian, Polish, Greek and Arabic [8]. Another service offers support services and counselling in different languages, such as Russian, Greek, Croatian, Italian and Turkish [12].

### Bavaria

For Bavaria four services were identified as supplying counselling and/or support services and one was found that trains multilingual dementia aides to offer culturally sensitive support services. These services address Turkish, Russian, Arabic, Italian, Greek, Serbo-Croat, Polish, Spanish and Vietnamese speaking people [8].

### Hesse

A total of five services that offer counselling and/or support and care services in Turkish or Russian were found [8].

### Berlin

In Berlin four services were found that provide counselling and/or support in Turkish, Serbo-Croat, Spanish, Russian, Arabic and Vietnamese [8].

### Saxony

A total of three counselling and care services that offer services in Russian and one also in Vietnamese were detected [8].

### Hamburg

In Hamburg two services were identified, one is a multilingual care facility that also offers home care and one is a counselling service in Turkish [8].

### Lower Saxony

There were two services found that offer counselling in Turkish, Russian and Italian [8].

### Bremen, Saarland, Schleswig-Holstein, Thuringia

One counselling (and support) service in different languages such as Turkish, Russian, Italian, Polish, Arabic and English could be found in each of these federal states [8].

### Brandenburg, Mecklenburg-Western Pomerania, Rhineland-Palatinate, Saxony-Anhalt

No services could be found [8].

## Discussion

The national dementia strategy aims to expand the availability of culturally sensitive information and healthcare services for PwM (with dementia), and this scoping review sets out to determine the number of these services and projects on dementia and migration that are available in Germany. Currently, most services of that kind are found in North Rhine-Westphalia, Baden-Wuerttemberg, and Bavaria and Hesse. Berlin, Bremen, Hamburg, Lower Saxony, Saarland, Saxony, Schleswig-Holstein and Thuringia provide fewer services each, while no services could be identified in Brandenburg, Mecklenburg-Western Pomerania, Rhineland-Palatinate and Saxony-Anhalt. This outcome mirrors in general the distribution of PwM with dementia in Germany, as a majority of the affected PwM live in the federal states that provide most services. Surprisingly, Rhineland-Palatinate, although the area has quite a large number of PwM with dementia, seems to provide no services, while for Lower Saxony, despite housing 7200 PwM with dementia, only 2 services could be found.

The majority of services for people with dementia from (a) Poland are located in Baden-Wuerttemberg, (b) Turkey are found in North Rhine-Westphalia, Baden-Wuerttemberg and Bavaria, (c) Russia are in Baden-Wuerttemberg, Bavaria, Berlin and Saxony, and (d) Italy are located in Baden-Wuerttemberg, Bavaria, Saarland and Lower Saxony. Although people with dementia from Romania are one of the largest groups of PwM with dementia in Germany, it was not possible to identify services for them.

These results indicate that some efforts have been made to provide information and support to PwM with dementia. Most of these services are located in the federal states where most of the PwM (with dementia) live. It seems to be necessary, however, to establish services in all federal states since every person has a right to person-centered care, which means that information and healthcare services should be available in every region and also be tailored to different ethnicities, as stated in the national dementia strategy [14]. Of the federal states four do not offer services, while six offer only one or two services. This means that a high proportion of PwM with dementia and their families cannot be adequately supported. Furthermore, most of the services offer counselling, and only a few are care facilities and home care services. This reveals a huge gap in care services that needs to be further investigated.

As described, due to the additional challenges PwM face, the usual mainstream services are not entirely suitable to take care of PwM. Different aspects and culturally sensitive measures are important considerations when informing PwM with dementia, as such approaches enable these individuals to gain access to the healthcare system [1, 3, 19, 20, 26]. The national dementia strategy highlights the importance of offering low-threshold services and adapting services to the needs of PwM with dementia; the Alzheimer Europe report on intercultural care (2018) [1], and Schmachtenberg et al. (2021) [26] pointed out how this can be implemented.

The projects that focus on dementia and migration show an effort to inform PwM with dementia and their families about dementia and healthcare services and to connect healthcare providers with one another to expand and improve healthcare for PwM with dementia and their families. Nevertheless, more projects are needed to a) identify PwM with dementia and their needs and demands regarding the healthcare system, its services and information and b) support PwM with dementia and their families in their situation in relation to the healthcare system, service providers and policy makers. When doing so, it is imperative to include the people affected to obtain first-hand information from them on what is needed [5]. Furthermore, it is not enough to have single services and projects that attempt to determine what is needed for this population. It is important to combine these separate services and projects with relevant stakeholders [14], such as people from the healthcare system, service providers, researchers and the people affected, to create access and services that improve the situation of PwM with dementia and their families. This is why initiatives that do exactly that, like DeMigranz, are important.

These results are to be taken with caution. The chosen search strategy resulted in a selective assortment; thus, it does not aim to give a conclusive overview. One should acknowledge that in routine care, there are many people engaged in improved care for PwM, and their efforts are often not published or presented to a broader audience. Therefore, there is the possibility that the “*Netzwerkkarte*” and the search presented herein may not include every healthcare service and project related to dementia and migration. One attempt to get a more detailed overview could be to set up an initiative similar to DeMigranz at the federal state or even regional level. For example, care providers and staff, healthcare professionals, representatives of local Alzheimer’s societies, dementia networks and psychiatric and medical associations, members of associations for patients and/or relatives could come together and have as a possible goal to identify the healthcare and information services in the region and to create a comprehensive overview of these services. In this way, it might be possible to look in more detail at what services are available. Another possibility would be to use the approach of the “EU-Atlas: Dementia & Migration”. Here, using various methods, an overview of the topic of dementia and migration, including the current care situation of those affected and available healthcare services, was created at the European level [21], which could be carried out in more detail at the German level. Furthermore, there is the possibility that some migrant groups are considered to be well integrated into society and the healthcare system and therefore no need might be seen to offer specialized services for them. Nevertheless, this review provides valuable information on the current state of culturally sensitive healthcare services and projects in Germany and fosters awareness and exchange.

## Practical conclusion

- This scoping review identified 48 culturally sensitive healthcare services and projects on dementia and migration in Germany.
- There are projects aiming at offering support and information to PwM with dementia and their families; however, more research and knowledge transfer is needed.
- Systematic availability and access are needed. Every federal state should offer tailored information and services for PwM with dementia and their families regardless of how many PwM with dementia live in the federal state. In addition, more projects on dementia and migration should be systematically funded, conducted and made available to others.

## Declarations

For this article no studies with human participants or animals were performed by any of the authors. All studies performed were in accordance with the ethical standards indicated in each case.
